# Reformation programmes in medium-security correctional facilities in Kwara State, Nigeria: evidence of functionality gaps

**DOI:** 10.3389/fresc.2026.1810028

**Published:** 2026-06-16

**Authors:** Muhinat B. Bello, Nimota Jibola Kadir Abdullahi, Mulikat L. Mustapha, Sonibare Segun

**Affiliations:** 1 Department of Social Sciences Education Faculty of Education, University of Ilorin, Ilorin, Nigeria; 2 Department of Educational Management Faculty of Education, University of Ilorin, Ilorin, Nigeria; 3 Department of Educational Guidance and Counselling Faculty of Education, University of Ilorin, Ilorin, Nigeria

**Keywords:** availability, correctional, inmate, recidivism, rehabilitation

## Abstract

This study examines the availability, adequacy, and functionality of reformation programmes in selected medium-security correctional facilities in Kwara State. The study adopts a descriptive ex-post facto design and utilises a researcher-developed checklist to document observable rehabilitation resources and activities across three custodial centres. Grounded in Bandura's(1977) Social Learning Theory. This study operationalises Social Learning Theory by linking the availability, adequacy, and functionality of reformation programmes to the extent to which correctional environments enable observational learning, behavioural modelling, and reinforcement processes among inmates. It theorizes that limited prosocial modeling through education and vocational training perpetuates recidivism due to inadequate resources and overcrowding. Data were analysed using descriptive statistics. Findings indicate that 67% of the facilities lacked functional educational programmes, while all facilities (100%) had functional religious programmes. Vocational training was present in all centres, but was fully functional in only one facility (33%). Overcrowding was observed in two of the three facilities (67%), significantly constraining programme delivery. Critical digital learning infrastructure, including computers and internet access, was absent or non-functional across all facilities (100%). These gaps limit opportunities for skill acquisition, structured learning, and behavioural development, thereby weakening inmates' prospects for successful reintegration. The study highlights the need for targeted investment in educational infrastructure, digital learning integration, and staffing to enhance the rehabilitative capacity of correctional institutions.

## Introduction

Correctional rehabilitation has become a central concern in contemporary criminal justice systems, reflecting a shift from purely punitive approaches toward strategies that support offender reintegration and reduce recidivism. Across different contexts, reformation programmes, such as education, vocational training, and psychosocial support, are increasingly recognised as essential components of effective correctional practice. Empirical evidence indicates that well-structured rehabilitation initiatives can improve post-release outcomes by enhancing employability, promoting behavioural change, and reducing the likelihood of re-offending (1–3).

In many low- and middle-income countries, however, the implementation of such programmes is often constrained by systemic challenges, including overcrowding, limited funding, and inadequate infrastructure. Nigeria presents a particularly relevant case, as correctional facilities are formally mandated to provide rehabilitation services but frequently operate under significant resource limitations. Existing studies suggest that while some institutions offer educational and vocational activities, access remains uneven and programme delivery is often inconsistent (4, 5). These conditions raise important questions about the actual functionality of reformation programmes within specific institutional settings. While these challenges are widely documented across Nigeria, this study specifically focuses on medium-security correctional facilities in Kwara State to provide context-specific empirical evidence.

International research highlights a range of approaches to correctional rehabilitation, with emphasis on evidence-based interventions, structured education systems, and skills development aligned with labour market demands (6). For example, studies in developed contexts show that access to educational programmes, including digital learning, can significantly improve rehabilitation outcomes and reduce recidivism (7–9). However, the direct transferability of these models to resource-constrained environments remains uncertain, as contextual factors such as infrastructure, staffing, and funding influence implementation.

Within the Nigerian context, several studies have documented persistent challenges affecting correctional effectiveness. Overcrowding, in particular, has been identified as a major constraint on the delivery of rehabilitation programmes, limiting inmates' access to available services and reducing programme effectiveness (10). Similarly, inadequate educational infrastructure, shortages of trained personnel, and limited access to learning materials have been shown to undermine rehabilitation efforts (11). In addition, vocational training programmes, where available, are often under-resourced and may not align with labour market demands, thereby limiting their impact on post-release employment opportunities (12).

Recent scholarship has increasingly emphasised the role of structured rehabilitation programmes in reducing recidivism and improving reintegration outcomes. Studies have shown that access to education and vocational training significantly enhances employability and post-release adjustment (10). In addition, digital learning interventions have emerged as critical tools for expanding access to correctional education, particularly in resource-constrained settings (7, 13). However, in sub-Saharan Africa, implementation remains uneven due to infrastructural and institutional constraints (14).

Despite these contributions, much of the existing literature focuses on national-level assessments or general policy discussions, with limited empirical evidence at the facility level. This creates a gap in understanding how reformation programmes are actually implemented and function within specific correctional institutions. Furthermore, while some studies reference international standards, there is limited systematic assessment of programme availability, adequacy, and functionality using clearly defined and observable indicators.

This study addresses this gap by focusing on selected medium-security correctional facilities in Kwara State. It provides a descriptive assessment of reformation programmes within these facilities, with particular attention to observable resources, programme availability, and operational status. By narrowing the scope to specific institutions, the study avoids broad generalisations and instead offers context-specific insights into current rehabilitation practices. The study is guided by the need to better understand the extent to which existing reformation programmes are present and functional within the selected facilities. Such an understanding is important for informing future research, improving programme design, and supporting evidence-based discussions on correctional reform in Nigeria.

Drawing on Social Learning Theory, this study conceptualises correctional rehabilitation as a function of environmental learning conditions within institutional settings. Specifically, the availability of programmes reflects the presence of learning opportunities, adequacy represents the sufficiency of exposure to prosocial models, and functionality captures the extent to which such programmes actively facilitate interaction, reinforcement, and behavioural engagement. This theoretical framing informs both the selection of variables and the interpretation of findings in this study.

### Statement of the problem

The effectiveness of correctional rehabilitation has become a critical concern in contemporary criminal justice systems, particularly in resource-constrained contexts. In Nigeria, medium-security correctional facilities are mandated to provide structured reformation programmes aimed at preparing inmates for reintegration into society. However, existing evidence suggests persistent challenges, including overcrowding, inadequate funding, and limited access to educational, vocational, and psychosocial support services, which may constrain the effectiveness of these programmes.

While international literature emphasises evidence-based and well-resourced rehabilitation models, there is limited empirical, facility-level data examining how such programmes are implemented and function within specific Nigerian contexts. In particular, few studies have systematically assessed the availability, adequacy, and functionality of reformation programmes within individual correctional facilities using clearly defined indicators.

This study addresses this gap by focusing on selected medium-security correctional facilities in Kwara State. It provides a descriptive assessment of existing reformation programmes within these facilities, with attention to observable resource provision and operational status. Rather than making broad generalisations about the national correctional system, the study aims to generate context-specific evidence that can inform further research and targeted policy discussions. By documenting current conditions at the facility level, the study contributes to a more grounded understanding of rehabilitation practices in Nigeria and highlights areas requiring further investigation, particularly in relation to programme implementation and resource allocation.

Bandura's (15) Social Learning Theory posits that behaviors are acquired via observation, imitation, and reinforcement from environmental models. In corrections, inmates learn prosocial skills through structured education/vocational programs providing positive role models; absent these, antisocial patterns persist, elevating recidivism.

### Purpose of the study

(a) Examine the reformation programmes available for inmates' reformation at medium security facilities in Nigeria?
(b) Find out the functionality of reformation programmes for the reformation of inmates in medium security facilities in Nigeria.
(c) How adequate are the reformation programmes for the reformation of inmates in medium security facilities in Nigeria?

### Research questions

1. What reformation programs (educational, vocational, religious) are available in selected medium-security correctional facilities in Kwara State?
2. How functional are the available reformation programs in these facilities?
3. How adequate are reformation programs relative to inmate population and facility capacity?

### Theoretical framework (social learning theory by albert bandura)

Albert Bandura's Social Learning Theory (1977) provides a strong sociological foundation for this study. The theory posits that individuals learn behaviours, values, and norms through observation, imitation, and interaction with their environment. In correctional rehabilitation, inmates' reformation depends significantly on their exposure to structured educational and vocational training, as well as positive role models within correctional facilities.

According to Bandura, behaviour is shaped by reinforcement and social modelling, meaning that if inmates are provided with opportunities for skill acquisition, education, and mentorship, they are more likely to adopt constructive behaviours that aid reintegration into society. However, the study's findings reveal that many correctional centers in Kwara State lack adequate educational and vocational training, limiting inmates' ability to learn positive behaviours and life skills. Without structured reformation programmes, inmates may resort to criminal behaviours upon release, reinforcing cycles of recidivism.

Applying Social Learning Theory, it becomes evident that correctional facilities must foster an environment where positive behaviours are modelled and reinforced. This includes providing trained educators, vocational trainers, and rehabilitative programs that encourage skill-building and self-improvement. By integrating this theory into policy reforms, correctional centers can create an environment that supports behavioural transformation, ensuring that inmates are better equipped for social and economic reintegration after incarceration.

In this study, Social Learning Theory is not only used as a conceptual reference but as an analytical lens guiding the assessment of correctional environments. The study assumes that rehabilitation outcomes are shaped by the extent to which institutional conditions enable consistent exposure to prosocial behaviours through structured programmes. Consequently, the variables of availability, adequacy, and functionality are treated as observable indicators of the strength or weakness of social learning conditions within the selected facilities.

### Methodology

#### Research design

This study adopted a descriptive ex-post facto design to assess the current state of reformation programmes in selected correctional facilities. The design was considered appropriate because the study did not involve manipulation of variables but rather focused on documenting and evaluating existing conditions within the facilities. The study was conducted in selected medium-security correctional facilities in Kwara State. The focus on Kwara State reflects the need for a facility-level assessment, rather than a broad national generalisation.

#### Population and sampling

The study population comprised staff of the Nigerian Correctional Service (NCoS) working within medium-security custodial centres in Kwara State. A purposive sampling technique was employed to select three medium-security correctional facilities (Oke-Kura, Mandala, and Omu-Aran), based on their accessibility and classification as medium-security institutions. Within each facility, relevant officials directly involved in inmate management and rehabilitation programmes (e.g., welfare officers, rehabilitation officers, and administrative staff) were selected. A total of 9 officials (3 per facility) participated in the study. These officials were chosen because of their direct knowledge of available programmes and facility operations. The use of purposive sampling was justified by the need to obtain informed responses from officials directly involved in rehabilitation programme implementation, thereby ensuring relevance and accuracy of data.

#### Instrument for data collection

Data were collected using a researcher-developed checklist, designed to systematically document the presence and condition of reformation programmes and facilities. The checklist was structured into three sections: (1) Availability of Programmes and Facilities, (2) Adequacy of Resources, and (3) Functionality of Programmes and Facilities. These constructs were theoretically informed by Social Learning Theory, which emphasises the role of environmental exposure and interaction in behavioural acquisition. Accordingly, the checklist was designed to capture not only the presence of programmes but also the extent to which they create functional conditions for observation, participation, and reinforcement of prosocial behaviours. To enhance clarity and replicability, the following operational definitions were adopted: Availability: Refers to the physical presence of a programme or facility within a correctional centre. It was measured dichotomously as: *Available (A)* or *Not Available (NA)*. Adequacy: Refers to the extent to which available resources (e.g., staff, materials, equipment) are sufficient relative to the inmate population. Adequacy was assessed based on observable ratios and categorised as:

*Adequate* or *Inadequate*. Functionality: Refers to whether a programme or facility is actively operational and being utilised by inmates at the time of data collection. It was measured as:

*Functional (F)* or *Not Functional (NF)*.

### Instrument development and validation

The checklist items were developed based on existing literature on correctional rehabilitation and commonly identified programme components, including educational, vocational, and religious activities. To ensure content validity, the instrument was reviewed by two experts in educational management and one expert in criminology, who assessed the relevance, clarity, and coverage of the items. Their feedback led to minor revisions in wording and structure. A pilot test was conducted in a correctional facility outside the study sample to assess clarity and usability. Based on the pilot results, ambiguous items were refined to improve consistency in responses. To enhance reliability, the checklist was applied consistently across all facilities using the same operational definitions, and observations were cross-verified with facility officials to minimise reporting bias.

### Data collection procedure

Data were collected through on-site visits to the selected facilities. The checklist was completed by the researcher in collaboration with the selected officials, who provided clarifications and access to facility information where necessary. Direct observation was also used to verify the presence and condition of facilities and programmes. Permission to conduct the study was obtained from the relevant authorities within the Nigerian Correctional Service. Participation of officials was voluntary, and no personal identifiers were recorded. Data were used strictly for academic purposes.

### Method of data analysis

Data collected were analysed using descriptive statistics, including frequencies and percentages. The results were presented in tables to show the distribution of responses across the three facilities. Given the exploratory and facility-specific nature of the study, the analysis was limited to descriptive interpretation, without inferential generalisation beyond the sampled institutions.

## Results

Data are presented according to the three research questions, using descriptive statistics (frequencies and simple proportions). Each table corresponds directly to a research question and reflects the operational definitions of availability, adequacy, and functionality as outlined in the methodology.

### Research question 1

#### What reformation programmes are available in selected medium-security correctional facilities in Kwara State?

Table 1 presents the availability of major reformation programmes across the three facilities. Educational programmes were only observed in Oke-Kura, while Omu-Aran and Mandala lacked formal educational provision. Vocational programmes were present in all facilities but were limited in scope in Omu-Aran and Mandala. Religious programmes were consistently available across all three centres. Overall, educational programmes were available in only one out of three facilities (33%), while vocational programmes were present in all facilities (100%) but limited in two (67%). Religious programmes were consistently available across all facilities (100%)

**Table 1:** Availability of reformation programmes across facilities.

Programme Type	Omu-Aran	Oke-Kura	Mandala
Educational Programmes	Not Available	Available	Not Available
Vocational Programmes	Limited Availability	Available	Limited Availability
Religious Programmes	Available	Available	Available

### Research question 2

#### How functional are the available reformation programmes in the selected facilities?

Table 2 shows that educational support facilities were largely non-functional across the three centres. Only Oke-Kura demonstrated limited functionality in terms of library access, textbooks, and trained personnel. Critical infrastructure, such as internet access and computers, was absent or non-functional across all facilities. Across all facilities, 100% of digital infrastructure components (internet and computers) were non-functional, while only one facility (33%) demonstrated partial functionality in educational resources.

**Table 2:** Functionality of selected educational and support facilities.

Facility Component	Omu-Aran	Oke-Kura	Mandala
Library	Not Functional	Functional	Functional
Textbooks	Not Functional	Functional	Functional
Writing Materials	Not Functional	Not Functional	Not Functional
Internet Access	Not Functional	Not Functional	Not Functional
Computers	Not Functional	Not Functional	Not Functional
Trained Teachers	Not Functional	Functional (Limited)	Not Functional

Table 3 indicates that vocational training programmes were more operational than educational programmes, particularly in Oke-Kura. However, functionality varied across facilities, with Omu-Aran showing fewer active programmes and Mandala demonstrating selective functionality. Tailoring and barbing were the most consistently functional skills across all centres.

**Table 3:** Functionality of vocational training programmes.

Vocational Skill	Omu-Aran	Oke-Kura	Mandala
Carpentry	Functional	Functional	Not Functional
Welding	Functional	Functional	Not Functional
Tailoring	Functional	Functional	Functional
Barbing	Functional	Functional	Functional
Electrical Works	Not Functional	Functional	Functional
Plumbing	Not Functional	Not Functional	Functional
Shoemaking	Not Functional	Functional	Functional

**Table 4:** Functionality of religious programmes.

Religious Programme	Omu-Aran	Oke-Kura	Mandala
Christian Activities	Functional	Functional	Functional
Islamic Activities	Functional	Functional	Functional

Religious programmes were fully functional across all facilities, representing the most consistently operational form of inmate engagement.

### Research question 3

#### How adequate are the available reformation programmes relative to inmate population?

Table 5 shows that two of the three facilities (Oke-Kura and Mandala) were operating far above their designed capacities, indicating significant overcrowding. Only Omu-Aran operated within its capacity. This imbalance has implications for the adequacy of available programmes.

**Table 5:** Capacity and inmate population of selected facilities.

Facility	Designed Capacity	Current Population	Status
Oke-Kura	121	547	Overcrowded
Mandala	200	456	Overcrowded
Omu-Aran	100	88	Within Capacity

Table 6 shows that educational programmes were inadequate across all facilities. Vocational programmes were only moderately adequate in Oke-Kura but inadequate in the other facilities. Religious programmes were the only category considered adequate across all centres.

**Table 6:** Adequacy of reformation programmes.

Programme Type	Omu-Aran	Oke-Kura	Mandala
Educational	Inadequate	Inadequate	Inadequate
Vocational	Inadequate	Moderately Adequate	Inadequate
Religious	Adequate	Adequate	Adequate

## Discussion

This study examined the availability, adequacy, and functionality of reformation programmes in selected medium-security correctional facilities in Kwara State. The findings provide facility-level evidence of how rehabilitation programmes are currently structured and implemented within the sampled institutions. Given the descriptive and context-specific nature of the data, the discussion is limited to interpretation within the studied facilities while drawing cautious connections to existing literature.

The findings indicate that educational programmes are either absent or minimally functional across most of the facilities studied. This observation is consistent with previous research highlighting limited access to structured educational opportunities within Nigerian correctional institutions (4, 16). The absence of key educational resources, such as trained instructors, learning materials, and basic infrastructure, suggests that educational rehabilitation may not be systematically prioritised in the sampled facilities. While prior studies have linked correctional education to improved reintegration outcomes, the present study does not directly measure post-release outcomes; therefore, it only highlights structural limitations in programme provision rather than their long-term effects. From a Social Learning Theory perspective, the limited availability and functionality of educational programmes suggest restricted opportunities for observational learning and cognitive skill development. Without structured educational engagement, inmates may lack access to positive role models and guided learning environments necessary for behavioural transformation.

Vocational training programmes were observed to be partially available but uneven in functionality across the facilities. In particular, one facility demonstrated relatively broader provision of vocational activities, while others showed limited or inconsistent implementation. This aligns with earlier findings that vocational training in Nigerian correctional settings is often constrained by inadequate tools, funding, and alignment with labour market demands (12). However, it is important to note that this study assessed only the presence and operational status of such programmes, not their quality or effectiveness in improving employability outcomes. The uneven functionality of vocational programmes further indicates inconsistent reinforcement mechanisms across facilities. Social Learning Theory suggests that behavioural change is strengthened through repeated practice and reinforcement; therefore, irregular or poorly resourced vocational activities may limit the internalisation of productive skills and behaviours.

A notable finding is the consistent availability and functionality of religious programmes across all sampled facilities. This supports existing literature suggesting that faith-based activities are among the most stable forms of inmate engagement in Nigerian correctional centres (4, 17). While religious programmes may contribute to moral and behavioural orientation, their dominance within the observed programme mix may also reflect institutional reliance on low-resource interventions, particularly in contexts where formal educational and vocational systems are underdeveloped. The consistent functionality of religious programmes may reflect the presence of stable social interaction and moral modelling environments. Within the framework of Social Learning Theory, such settings can facilitate value-based learning; however, their impact may be limited if not complemented by structured skill-based and educational interventions.

The study also highlights the potential influence of overcrowding on programme adequacy, particularly in facilities operating significantly above their designed capacity. This observation is consistent with prior research indicating that overcrowding limits access to available resources and reduces the effectiveness of rehabilitation efforts (10, 13). In the present study, overcrowded facilities appeared to experience greater constraints in programme delivery, although causal relationships cannot be established due to the descriptive design. Overcrowding may also be interpreted as a structural constraint on social learning processes, as it reduces access to programmes and limits meaningful interaction between inmates and facilitators. This weakens the environmental conditions necessary for effective behavioural modelling and reinforcement.

Furthermore, the absence of digital learning infrastructure, including internet access and computers, was evident across all facilities. This finding reflects broader challenges identified in the literature regarding the limited integration of technology in correctional education in developing contexts. Studies in other settings have suggested that digital learning can enhance access to education and support rehabilitation (7), but the current study indicates that such approaches are not yet operational within the sampled facilities.

While the study references international perspectives on correctional rehabilitation, it does not attempt to directly evaluate compliance with global standards. Instead, the findings highlight observable gaps in programme provision that may be relevant for future comparative or policy-oriented research. Recent studies have emphasised the importance of context-sensitive approaches to correctional reform, particularly in resource-constrained environments (14, 18, 19). In this regard, the present study contributes empirical evidence that may support further investigation into how rehabilitation programmes can be strengthened within similar institutional contexts. The findings suggest that reformation programmes within the studied facilities are characterised by limited educational provision, uneven vocational implementation, and reliance on religious activities, alongside structural constraints such as overcrowding and inadequate resources. However, these observations are restricted to the sampled facilities and should not be generalised beyond the study context.

From a policy perspective, these findings underscore the urgent need for targeted correctional reforms in Nigeria. Investment in educational infrastructure, digital learning technologies, and skilled personnel is critical for enhancing rehabilitation outcomes. Furthermore, addressing overcrowding through alternative sentencing and facility expansion could improve access to reformation programmes. Policymakers should prioritise evidence-based rehabilitation strategies that integrate vocational relevance with labour market demands to strengthen post-release reintegration

## Conclusion

This study examined the availability, adequacy, and functionality of reformation programmes in selected medium-security correctional facilities in Kwara State. The findings provide facility-level insights into the current state of rehabilitation practices within the sampled institutions.

The results indicate that reformation programmes are unevenly distributed and variably functional across the facilities studied. Educational programmes were largely absent or minimally operational, while vocational training was present but inconsistently implemented and often constrained by limited resources. In contrast, religious programmes were consistently available and functional across all centres. The findings also suggest that structural factors, particularly overcrowding and resource limitations, may influence the adequacy and delivery of available programmes. Given the descriptive scope and limited sample size, the study does not generalise beyond the selected facilities. Interpreted through Social Learning Theory, these findings suggest that the limited availability, adequacy, and functionality of reformation programmes constrain the capacity of correctional environments to support sustained behavioural learning and transformation. The study demonstrates that significant structural and operational gaps persist in the delivery of reformation programmes within the sampled facilities. Addressing these gaps requires urgent and coordinated policy action focused on expanding educational access, integrating digital learning systems, improving staffing, and reducing overcrowding. Without such interventions, the rehabilitative potential of correctional institutions will remain limited, with implications for recidivism and public safety

### Recommendations

Based on the findings of this study, several recommendations are proposed to improve the provision and delivery of reformation programmes in the selected medium-security correctional facilities in Kwara State. In line with Social Learning Theory, improving correctional rehabilitation requires strengthening the environmental conditions that support observation, interaction, and reinforcement of prosocial behaviours.

First, there is a need to strengthen the provision of educational programmes within the facilities studied. The findings indicate that educational opportunities are either absent or minimally functional, suggesting the importance of improving access to structured learning through the provision of basic instructional materials, the deployment of qualified teaching personnel, and the organisation of regular educational activities for inmates.

In addition, vocational training programmes should be enhanced to improve their operational capacity. While vocational activities were present in some facilities, their effectiveness appeared to be constrained by limited tools, equipment, and materials. Improving these resources, as well as aligning vocational training with locally relevant and marketable skills, may increase the practical value of such programmes within the correctional context.

Furthermore, the observed limitations in infrastructure and programme delivery point to the need for improved resource allocation. Addressing shortages in facilities, learning materials, and staffing may contribute to more effective implementation of reformation programmes within the studied institutions.

The issue of overcrowding, particularly in some of the facilities examined, also requires attention. The imbalance between designed capacity and inmate population may limit access to available programmes and reduce their overall effectiveness. Efforts aimed at managing inmate population levels could therefore support improved access to rehabilitation activities.

There is also a need to establish more systematic mechanisms for monitoring and evaluating reformation programmes. Regular assessment of programme availability, adequacy, and functionality would provide a basis for identifying gaps and informing incremental improvements in programme delivery.

Finally, further research is recommended to build on the findings of this study. Future studies could adopt larger and more diverse samples, as well as mixed-method approaches, to examine not only the presence of rehabilitation programmes but also their quality, utilisation, and potential outcomes in different correctional settings.

## Data Availability

The original contributions presented in the study are included in the article/Supplementary Material, further inquiries can be directed to the corresponding author.
